# An Ultrasound Image-and-Intervention Paradigm for a Neglected Lumbar Transverse Process Fracture: A Case Report of Diagnosis and Hydrodissection Treatment

**DOI:** 10.7759/cureus.98190

**Published:** 2025-11-30

**Authors:** Yonghyun Yoon, King Hei Stanley Lam, Jaeyoung Lee, Jaeik Choi, Gyung Seog KO, Teinny Suryadi, Anwar Suhaimi, Daniel Chiung-Jui Su

**Affiliations:** 1 Orthopedic Surgery, Hallym University Kangnam Sacred Heart Hospital, Seoul, KOR; 2 Orthopedics, Incheon Terminal Orthopedic Surgery Clinic, Incheon, KOR; 3 Faculty of Medicine, The University of Hong Kong, Hong Kong, HKG; 4 The Board of Clinical Research, The Hong Kong Institute of Musculoskeletal Medicine, Kowloon, HKG; 5 Faculty of Medicine, The Chinese University of Hong Kong, New Territories, HKG; 6 Rehabilitation Medicine, Dr. Choi's Rehabilitation and Medicine, Kyengido, KOR; 7 Orthopedics, Ko GyungSeog Orthopaedic Clinic, Seoul, KOR; 8 Physical Medicine and Rehabilitation, Synergy Clinic, Jakarta, IDN; 9 Physical Medicine and Rehabilitation, Hermina Podomoro Hospital, Jakarta, IDN; 10 Rehabilitation Medicine, University Malaya Medical Centre, Kuala Lumpur, MYS; 11 Rehabilitation Medicine, University Malaya, Kuala Lumpur, MYS; 12 Physical Medicine and Rehabilitation, Chi Mei Medical Center, Tainan, TWN

**Keywords:** chronic low back pain, dextrose prolotherapy, hydrodissection, intertransversarii muscles, lumbar transverse process fracture, musculoskeletal ultrasound, referred groin pain, regenerative injection therapy, sonoguided digital palpation, stress fracture

## Abstract

Lumbar transverse process (TP) fractures are often overlooked, leading to chronic pain that is frequently refractory to standard treatments. This case report describes the diagnostic use of musculoskeletal ultrasound (MSK-US) and a novel therapeutic application of ultrasound-guided hydrodissection for a case of neglected TP stress fracture.

A 41-year-old male presented with an 18-month history of chronic right groin and thoracolumbar pain, unresponsive to extensive prior treatments, including physical therapy, medications, and multiple nerve blocks. MSK-US confirmed a cortical irregularity and step-off deformity at the left L2 TP, with sonoguided digital palpation precisely localizing the source of his symptoms. An ultrasound-guided hydrodissection procedure was performed using 5% dextrose in water (D5W) to address suspected neurovascular entrapment.

The patient reported an immediate 80% reduction in resting pain following the procedure. After two additional weekly sessions, he achieved a sustained 95% pain reduction and complete resolution of physical examination deficits. Follow-up imaging demonstrated evidence of bony healing.

This case highlights MSK-US as a pivotal diagnostic tool for elusive chronic TP fractures and introduces ultrasound-guided hydrodissection as a novel, minimally invasive therapeutic strategy. This "image-and-intervene" paradigm effectively provided rapid pain relief, functional restoration, and facilitated a healing environment, offering a promising approach for managing similar refractory chronic pain conditions.

## Introduction

Low back pain (LBP) represents one of the most prevalent and disabling musculoskeletal conditions worldwide, with a lifetime prevalence exceeding 80% and a significant socioeconomic burden due to healthcare costs and lost productivity [1,2]. While the majority of LBP cases are non-specific, establishing a precise anatomical diagnosis is crucial for effective management, particularly in cases refractory to conventional therapy [3,4]. Vertebral fractures are a well-documented cause of LBP, typically associated with high-energy trauma in younger populations or osteoporosis in the elderly [5,6]. The diagnostic focus in such cases is often on the integrity of the anterior and middle spinal columns, as reflected in comprehensive systems such as the AO Spine and Denis classifications, which address vertebral body compression, burst fractures, and fracture-dislocations [7,8]. Consequently, fractures of the posterior elements, particularly the transverse processes (TPs), are frequently marginalized within these diagnostic and therapeutic schemas [9,10].

The lumbar transverse processes serve as critical biomechanical lever arms and attachment sites for major stabilizing muscles of the core and pelvis, including the quadratus lumborum, psoas major, and the intertransversarii [11,12]. TP fractures typically result from direct trauma or, more commonly, from violent muscular contraction and torsional forces transmitted through these muscular attachments [13,14]. The standard diagnostic algorithm for suspected TP fracture following trauma traditionally includes radiography, followed by computed tomography (CT) for confirmation [15,16]. However, the sensitivity of plain radiographs for detecting these fractures, especially minor or non-displaced ones, is notoriously poor, reported to be as low as 50%, leading to a high rate of initial oversight [17,18]. In contrast, CT boasts a sensitivity and specificity nearing 95-100% for bony detail and is rightly considered the gold standard for acute fracture diagnosis [19,20]. Magnetic resonance imaging (MRI) excels in visualizing bone marrow edema, disc pathology, and soft tissue injuries but is less specific for cortical disruption and is not routinely indicated for isolated posterior element pain without neurological signs [21,22]. Despite the accuracy of CT and MRI, their utilization is often constrained in cases of low-energy trauma or chronic, unexplained pain due to factors of cost, accessibility, time, and, in the case of CT, radiation exposure [23,24]. This frequently results in chronic TP fractures remaining undiagnosed for extended periods, becoming a hidden source of persistent pain and functional disability [25,26].

Musculoskeletal ultrasound (MSK-US) is an imaging modality whose applications are rapidly expanding beyond its traditional realms of tendon and joint evaluation [27,28]. Its distinct advantages include real-time dynamic imaging, a lack of ionizing radiation, high patient tolerance, and the unique ability to correlate anatomical findings with precise pain localization through sonopalpation [29,30]. MSK-US has an established role in guiding interventions for spinal conditions, such as facet joint injections and medial branch blocks [31,32]. Recently, several case series have reported on the promising use of ultrasound for diagnosing acute lumbar TP fractures [33,34], proposing methods that utilize both high-resolution static evaluation of the cortical bone and dynamic assessment during patient movement. However, these applications have been confined to acute post-traumatic settings, with subsequent management strategies limited to conservative measures like rest and analgesia.

For chronic, neglected fractures where pain persists despite bony stability, the underlying pathophysiology often shifts to surrounding soft tissue fibrosis and neurovascular entrapment within the muscular attachments [35]. This creates a significant therapeutic gap that demands innovative, minimally invasive strategies capable of modulating the local tissue microenvironment to break the cycle of pain and facilitate healing. In this context, musculoskeletal ultrasound (MSK-US) offers a promising tool, not only for diagnosis but also for guiding targeted interventions. The capability of ultrasound to directly visualize cortical breach and step-off is particularly advantageous for fracture detection, and its clinical utility across various bony and osteochondral injuries continues to expand [36,37]. A 2021 systematic review on foot and ankle fractures further supports its clinically adequate sensitivity and specificity when proper examination protocols are employed [38]. Furthermore, ultrasound-guided hydrodissection has emerged as a potential strategy to address soft tissue fibrosis, utilizing fluid pressure to mechanically dissect fibrotic tissue planes and release entrapped structures. The use of an injectate such as 5% dextrose in water (D5W) is grounded in its proposed dual mechanism of action: immediate mechanical dissection and potential long-term neuromodulation and tissue regeneration through osmotic and pro-sclerotic effects [39,40]. This case report describes the application of a combined "image-and-intervene" paradigm for this challenging condition.

## Case presentation

A 41-year-old male office worker, right-hand dominant, presented to our musculoskeletal medicine clinic with chief complaints of chronic, debilitating right-sided thoracolumbar back pain and right groin discomfort. The patient had no significant past medical history prior to the inciting injury.

History of present illness 

The patient's symptoms began approximately 18 months prior to presentation following a traumatic incident during a recreational basketball game. He sustained a direct impact to his right lateral thorax during a collision with another player, resulting in immediate, sharp pain. Evaluation at a local emergency department included a radiographic rib series, which confirmed an acute, non-displaced fracture of the right sixth rib. He managed conservatively with relative rest and over-the-counter analgesics (NSAIDs). While the acute rib pain subsided over several weeks, he subsequently developed a persistent, deep, aching pain in his right dorsal lumbar region, which radiated anteriorly into the right groin. This pain was exacerbated by trunk rotation, bending, and prolonged sitting.

Over the following year, he sought care from multiple providers. He underwent several months of physical therapy focusing on core stabilization and manual therapy, which yielded minimal improvement. A pharmacological regimen including NSAIDs (Naproxen 500mg BID) and a weak opioid (Tramadol 50mg PRN) provided only transient, partial relief. Due to the persistent and puzzling nature of his pain, he was referred to a university hospital's pain management service and subsequently to a private pain specialty center. Over a six-month period, he underwent a series of approximately 10 interventional procedures, including intercostal nerve blocks (at T6-T8 levels over the right side) and the right thoracic paravertebral blocks (TPVB). These interventions provided no significant or lasting relief. The persistent neuropathic quality of his right groin pain led a consultant to suspect post-herpetic neuralgia, despite the absence of a documented shingles rash. A Varicella Zoster Virus (VZV) IgG antibody test returned positive, and he subsequently received a course of gabapentin and a trial of pregabalin for suspected zoster-related neuralgia. This treatment also failed to alleviate his symptoms. His pain continued to worsen, severely impacting his sleep, ability to exercise, and overall quality of life, leading to the onset of secondary depressive symptoms.

Physical examination

Upon presentation to our clinic, the patient appeared fatigued but in no acute distress. No antalgic gait was observed, and he could perform all activities of daily living independently, though with evident discomfort.

Inspection

No visible swelling, erythema, or muscle atrophy was noted in the thoracolumbar region or lower extremities.

Palpation

Significant focal tenderness was elicited upon deep palpation over the left L2 transverse process, posteriorly. This finding was notably contralateral to his subjective complaints of right dorsal low back pain and right groin pain. No tenderness was noted over the spinous processes or sacroiliac joints.

*Range of Motion (ROM)* 

Active lumbar flexion was 80% of normal, and extension was 50%. However, lumbar rotation and side-bending to the right were markedly limited to less than 50% of the expected range and reproducibly elicited his familiar right dorsal low back pain and right groin pain.

Neurological examination

*Sensory* 

Light touch and pinprick testing revealed allodynia and hypersensitivity in the right inguinal area (L1-L2 dermatome).

Motor

Manual muscle testing revealed pain-associated weakness (graded 4/5 on the Medical Research Council scale) of the left hip flexors (iliopsoas). Strength in all other major muscle groups of the lower extremities was normal (5/5).

Reflexes

Patellar and Achilles deep tendon reflexes were normal and symmetric.

Special Tests

The straight leg raise test (SLRT) was negative bilaterally. Faber's test and the femoral nerve stretch test were also negative.

Diagnostic imaging 

Based on the intriguing finding of left-sided tenderness and weakness in a patient with right-sided pain, a focused radiographic evaluation was initiated.

*X-ray* 

**Figure 1:**
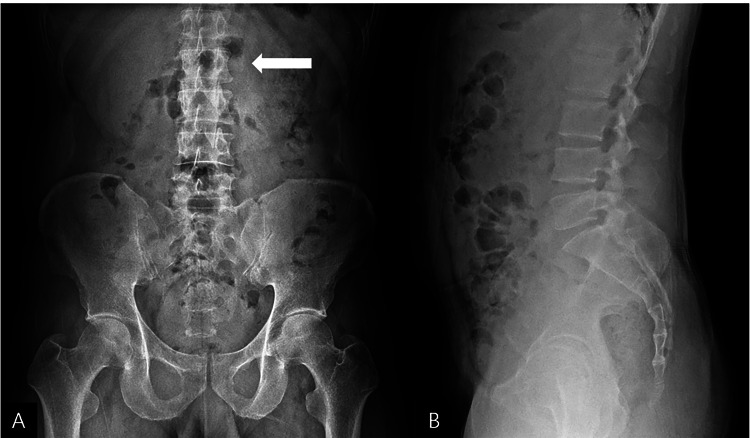
X-ray imaging of lumbar spine with identified fracture Lumbar spine X-ray. (A) AP View: No definite fracture line is present; however, subtle cortical irregularities at the tip of the left L2 transverse process are noted (arrow). (B) Lateral View: No significant vertebral body fracture or degenerative changes are observed.

Radiographs of the thoracolumbar spine were obtained. The lumbar spine X-ray (AP view) only revealed subtle cortical irregularity with no obvious radiolucent line at the tip of the left L2 transverse process (Figure 1A). No other vertebral body fractures or significant degenerative changes were noted (Figure 1B). A separate rib series (not shown) confirmed an old, healed fracture of the right sixth rib.

Ultrasonographic Evaluation and Sonoguided Digital Palpation (SDP)

A dedicated diagnostic ultrasound examination was performed, which integrated high-resolution static imaging with sonoguided digital palpation (SDP). SDP is a technique that combines the principles of osteopathic layered palpation with dynamic real-time ultrasound visualization, allowing for the precise anatomical correlation of palpatory findings and pain reproduction.

*Equipment and Settings* 

A curvilinear transducer (2-5 MHz) was used. The depth was set to 6 cm with the focal zone positioned at the cortical interface; tissue harmonic imaging was activated, and spatial compounding was deactivated to optimize bony detail. Color/power Doppler sweeps were performed to identify and avoid segmental vessels.

*Static Exam* 

**Figure 2:**
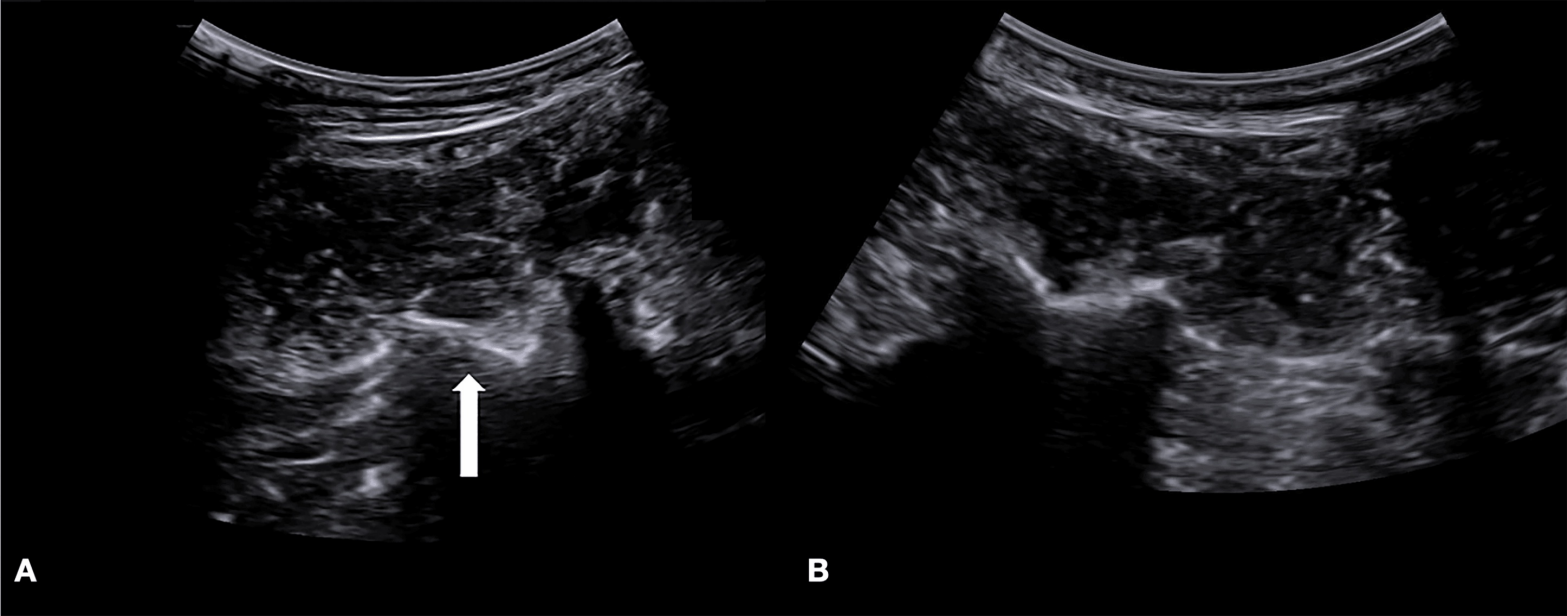
Ultrasound evaluation of transverse processes Comparative ultrasound evaluation of transverse processes (A) Fractured left L2 transverse process, demonstrating cortical disruption, irregularity, and a step-off deformity (indicated by the arrow). (B) Normal right L2 transverse process, displaying a smooth, continuous hyperechoic cortical line.

With the patient in a prone position, the L2 level was confirmed by rib counting from T12. On the left side, the L2 transverse process (TP) showed a distinct cortical step-off and irregularity (Figure 2B) compared with the smooth, continuous hyperechoic cortical line of the contralateral, normal L2 TP (Figure 2A).

Sonoguided Digital Palpation (SDP)

The examination continued with the patient in a side-lying (lateral decubitus, right side down) position to facilitate dynamic assessment. SDP was considered positive when (i) the patient’s familiar right-sided dorsal lumbar/groin pain pattern was reproduced and (ii) the pain intensity increased by ≥2 points on the Numeric Rating Scale (NRS). Three specific maneuvers were performed:

Method 1 (probe-driven lateral→medial shearing): With the probe centered over the left L2 TP cortical step-off, graded pressure (G1→G2) was applied while gliding the probe from anterolateral to posteromedial to impart a controlled shearing force across the cortex. This maneuver consistently reproduced the patient’s right dorsal lumbar (back) pain (e.g., NRS 7/10; Video 1).

**Video 1:** Probe-driven shearing pressure technique for L2 transverse process evaluation Demonstration of method 1—probe-driven anterolateral-to-posteromedial shearing pressure. The ultrasound probe was centered over the left L2 transverse process cortical step-off. Graded pressure (G1→G2) is applied while gliding anterolaterally to posteromedially, imparting a controlled shearing force across the cortex. This technique consistently reproduced the patient’s right dorsal lumbar (back) pain.

Method 2 sonoguided digital palpation of left L2 transverse process; posteromedial→anterolateral direction: With the probe fixed over the osseous defect, the examiner applied graded posteromedial-to-anterolateral layered palpation along the line of the TP. This preferentially reproduced the patient’s right dorsal lumbar pain (Video 2).

**Video 2:** Sonoguided digital palpation of the left L2 transverse process (posteromedial to anterolateral) Demonstration of method 2— sonoguided digital palpation of the left L2 transverse process (with the digit pressure applied posteromedial to anterolateral) with the probe fixed. The ultrasound probe was maintained over the defect while the examiner applied posteromedial-to-anterolateral layered palpation (G1→G2) along the transverse process line. This maneuver preferentially reproduced the patient’s right dorsal lumbar pain.

Method 3 sonoguided digital palpation of the left L2 transverse process; (anterolateral→posteromedial direction): In the same position, graded anterolateral-to-posteromedial layered palpation was applied to the left L2 transverse process, with the probe fixed. This maneuver specifically reproduced the patient’s right groin pain (Video 3).

**Video 3:** Sonoguided digital palpation of the left L2 transverse process: anterolateral→posteromedial direction Demonstration of method 3—sonoguided digital palpation of the left L2 transverse process: with the probe fixed over the L2 transverse process. While maintaining the probe in the same position, digital layered palpation from the anterolateral to posteromedial direction (G1→G2) was applied. This maneuver reproduced the patient’s right groin pain.

Across all three SDP methods assessing the left L2 transverse process, the same contralateral (right-sided) pain pattern was consistently elicited, supporting the left L2 TP lesion as the pain generator for right dorsal lumbar and right groin pain. Palpation 1-2 cm away from the left L2 TP defect did not reproduce the index pain, indicating that a more superficial soft tissue source is unlikely.

Dynamic examination: maintaining the probe over the left L2 TP defect in the side-lying position with the right side down, the patient performed gentle trunk rotation and side-bending. No gross instability of the TP fragment was visualized, suggesting a healed but symptomatic chronic lesion (Video 4).

**Video 4:** Dynamic examination of left L2 transverse process stability with the patient rotating and side-bending Dynamic examination with the probe maintained over the left L2 transverse process defect while the patient was in a side-lying position with the right side down. The patient performed gentle trunk rotation and side-bending. No gross instability of the transverse process fragment was visualized, suggesting a healed but symptomatic chronic lesion.

Interpretation and Transition to a Dynamic Exam

Across all three methods, the same contralateral (right-sided) pain pattern was reproduced, supporting segmental-convergence-mediated contralateral referral and implicating the left L2 lesion as the generator. The dynamic examination was then performed in the same side-lying position with the probe maintained over the defect, and no gross instability was observed.

Although symptoms were perceived on the right, SDP over the left L2 TP reproduced the index pain, consistent with segmental convergence and contralateral referral. The rapid analgesic response after targeted hydrodissection supports the left-sided lesion as the true generator.

Based on these sonographic findings and the positive SDP, which precisely localized the source of his referred pain, a targeted therapeutic intervention was planned. The clinical presentation and imaging were suggestive of neurovascular entrapment and soft tissue fibrosis within the intertransversarii muscles, perpetuating the chronic pain cycle. This created a therapeutic gap that demanded a minimally invasive strategy capable of modulating the local tissue environment. We therefore introduced a novel therapeutic rationale for this condition: ultrasound-guided hydrodissection of the intertransversarii muscles. This technique utilizes fluid pressure to mechanically dissect fibrotic tissue planes and release entrapped structures. Furthermore, we employed 5% dextrose in water (D5W) as the injectate, a choice grounded in its proposed dual mechanism of action: immediate mechanical dissection and potential long-term neuromodulation and tissue regeneration through osmotic and pro-sclerotic effects [39,40]. The lesion's clinical relevance was substantiated through the pain mapping via SDP and the immediate analgesic response during a subsequent diagnostic/targeted hydrodissection. These findings, complemented by the absence of instability on dynamic scanning, justified this tissue-focused approach and established a combined 'image-and-intervene' paradigm for this challenging condition.

Intervention: Ultrasound-Guided Hydrodissection

**Figure 3:**
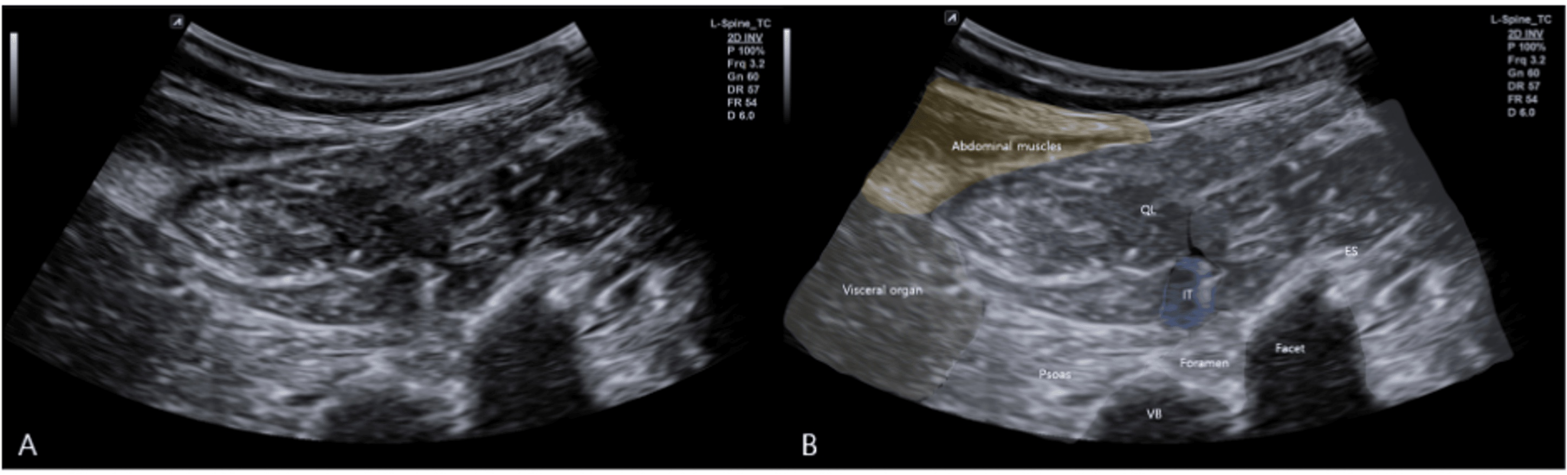
Right lateral decubitus ultrasound setup for intertransversarii assessment Right lateral decubitus setup and lateral acoustic window (A) Two-dimensional ultrasound image in the right lateral decubitus position, displaying the left L1–2 intertransversarii. (B) Schematic representation of anatomy and probe orientation for the lateral window targeting the intertransversarii fascial planes. ES: Erector Spinae; IT: Intertransversarii; QL: Quadratus Lumborum; VB: Vertebral Body.

The therapeutic procedure was performed with the patient in the right lateral decubitus position. Using an in-plane posterior-to-anterior approach under real-time ultrasound guidance, hydrodissection was carried out along the L1-2 intertransversarii fascial plane adjacent to the left L2 TP (Figure 3) to separate various layers of the thoracolumbar fasciae. Color/power Doppler was used pre-procedurally to map segmental vessels, and the pleural line was maintained at a safe distance.

**Figure 4:**
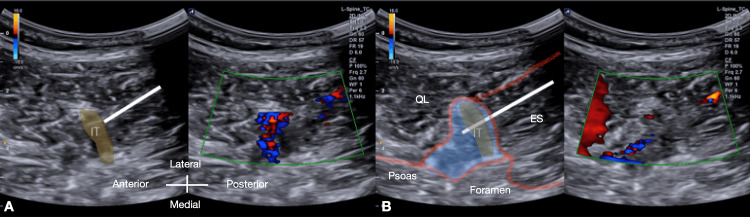
Live dual-view hydrodissection of the left L1/2 intertransversarii in right-side-down lateral decubitus position Live dual-view hydrodissection of the left L1/2 Intertransversarii in the right lateral decubitus position (A) In-plane posterior-to-anterior needle trajectory (indicated by the white straight line) approaching the intertransversarii plane (shown by the brownish shading). (B) Hydrodissection of various layers of thoracolumbar fascial planes (denoted by the red lines) with D5W spread (indicated by the blue shading). ES: Erector Spinae; IT: Intertransversarii.

Real-time hydrodissection was confirmed before incremental aliquots of D5W were delivered (Figure 4, Video 5).

**Video 5:** Real-time hydrodissection of the left L1/2 intertransversarii plane in right lateral decubitus position Demonstration of real-time hydrodissection in the right lateral decubitus position. The in-plane posterior-to-anterior needle trajectory is shown as it approaches the intertransversarii plane. Hydrodissection of the various layers of the thoracolumbar fasciae is visualized with D5W spread, highlighting the effectiveness of the technique.

Injectate

A total of 60 ml of 5% dextrose in water (D5W) was administered-30 ml above and 30 ml below the TP in the intertranversarii planes. D5W was chosen for its proposed dual neuromodulatory and mechanical hydrodissecting properties.

Immediate Outcome

During the injection, the patient reported a sensation of pressure followed by a familiar "tingling" radiating to his right groin. Re-provocation at the injection site during fluid delivery reproduced this sensation, which subsided as hydrodissection developed, further strengthening the lesion-symptom link. Immediately following the procedure, he reported an approximately 80% reduction in his resting pain. Reassessment showed markedly improved lumbar rotation and side-bending, and retesting of left hip flexor strength demonstrated immediate improvement to 5/5 without pain.

Follow-Up and Outcome

The patient underwent two additional hydrodissection treatments at the same site at one-week intervals. After the third session, he reported a sustained reduction in his pain, with NRS scores improving from 8 at rest to 1 and from 9 during rotation to 1. All previous positive physical exam findings, including pain-limited rotation, side-bending, and left hip flexor weakness, had completely resolved. He resumed all normal activities, including exercise.

**Figure 5:**
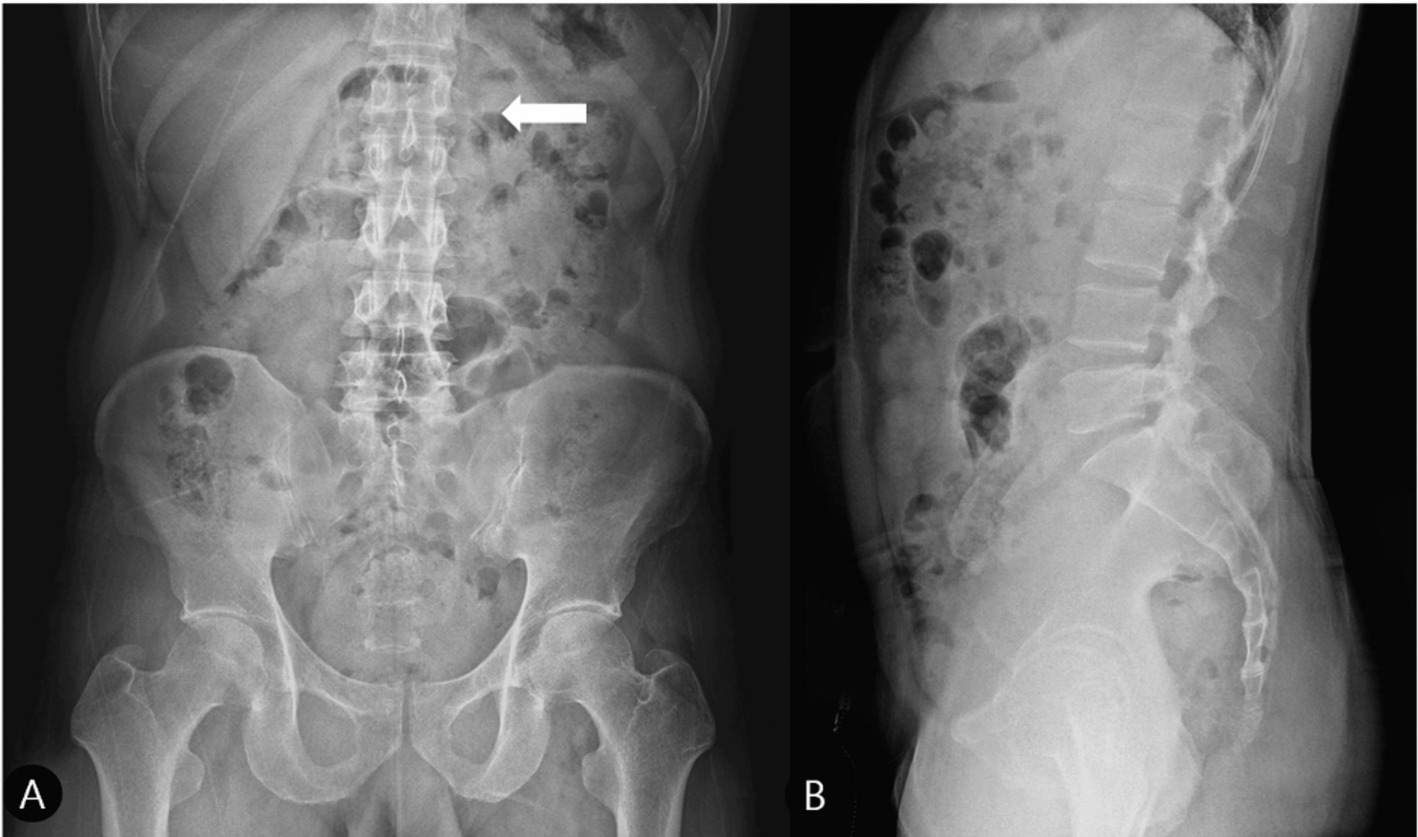
One-month follow-up lumbar X-ray One-month follow-up lumbar X-ray (AP view [A], lateral view [B]). Bridging callus and improved alignment at the left L2 transverse process are observed (solid arrow). Notable improvement in spinous process alignment is evident compared to baseline.

A follow-up X-ray obtained one month after the initial treatment showed improved cortical alignment and bridging callus formation at the left L2 transverse process fracture site (Figure 5).

The primary outcome was NRS at rest and during trunk rotation. Secondary outcomes included lumbar rotation/side-bending (% of normal), left hip-flexor MMT, sleep disturbance, and weekly rescue analgesic use. No adverse events occurred. Table 1 shows the time course. 

**Table 1:** Time-course of primary and secondary outcomes following intervention Summary of primary and secondary outcomes over time. The primary outcome measured is the Numeric Rating Scale (NRS) at rest and during trunk rotation. Secondary outcomes include lumbar rotation and side-bending as a percentage of normal, left hip-flexor manual muscle testing (MMT), sleep disturbance, and weekly rescue analgesic use. No adverse events were reported during the study. The time course illustrates changes from baseline through follow-up at 1-, 2-, and 4-weeks post-intervention.

Outcome	Baseline	Immediate post-#1	1 week (#2)	2 weeks (#3)	4 weeks (FU)
NRS – Rest (0–10)	8	2	2	1	1
NRS – Rotation (0–10)	9	3	2	1	1
Lumbar rotation (% of normal)	55	65	75	90	100
Lumbar side-bending (% of normal)	45	60	70	85	100
Hip flexor MMT (left, 0–5)	4/5†	5/5	5/5	5/5	5/5
Sleep disturbance (Y/N)	Y	N	N	N	N
Rescue analgesics / week	as needed	↓	↓	↓	0 (none)
Adverse events	–	–	–	–	–

## Discussion

This case report presents the novel application of diagnostic musculoskeletal ultrasound for identifying a neglected lumbar transverse process stress fracture and, more significantly, describes the successful use of ultrasound-guided hydrodissection of the intertransversarii muscles as a therapeutic intervention. To our knowledge, this represents the first report to detail this combined diagnostic and therapeutic approach for a chronic, post-traumatic TP stress fracture that proved refractory to an extensive array of conventional treatments, including multiple peripheral nerve blocks.

The diagnosis of lumbar TP fractures remains a persistent clinical challenge, particularly in the setting of low-energy trauma or chronic pain where a high degree of clinical suspicion is required [17,41]. As demonstrated in this case and supported by the existing literature, plain radiographs have poor sensitivity, and these fractures can be easily missed on initial evaluation [18]. While computed tomography (CT) is the undisputed gold standard for acute bony injury [19,20], its application is often limited in chronic, overlooked cases due to perceived low diagnostic yield, cost, and radiation exposure [23,24]. Magnetic resonance imaging (MRI) excels at visualizing bone marrow edema, a hallmark of acute or active stress fractures; however, in a chronic, stabilized stress fracture with a cortical step-off such as the one presented here, the initial edema may have resolved, leaving only the morphological cortical disruption, which MRI is less specific for than CT or high-resolution ultrasound [21,22]. Diagnostic ultrasound effectively bridges this gap by providing dynamic, high-resolution, and cost-effective visualization of the cortical bone contour, allowing for the direct identification of fracture lines, step-off deformities, and callus formation, as seen in our patient [33,34]. The unique ability to perform real-time SDP [42] to precisely localize the source of pain adds a functional component to the diagnosis that static imaging modalities like CT and MRI inherently lack [29,30]. This functional correlation was critical in our case, as the pain was referred contralaterally, a finding that would have confounded a diagnosis based on imaging alone. The expanding role of ultrasound in bone and osteochondral injury is supported by its growing application across various skeletal pathologies [36,37], with a 2021 systematic review further affirming its clinically adequate sensitivity and specificity for foot and ankle fractures when proper protocols are employed [38].

Lumbar transverse process fractures are often underdiagnosed, and depending on the mechanism of trauma, may indicate associated intra‑abdominal or visceral injuries, highlighting the need for careful and comprehensive assessment [43].

Previous literature on ultrasound for TP fractures has predominantly focused on its role in the acute trauma setting, often within emergency departments [33, 34]. Our case significantly expands this application to the chronic phase, demonstrating that ultrasound can not only identify the morphological sequelae of an old fracture (cortical irregularity, callus) but also confirm its ongoing clinical relevance through SDP. Furthermore, we introduced a dynamic component to the ultrasound assessment to evaluate for mechanical instability, which can directly inform treatment decisions and activity modifications. The stable appearance on the dynamic scan supported the use of a minimally invasive intervention rather than strict immobilization. Beyond emergency settings [33,34], practical reports from orthopedic outpatient clinics have demonstrated that ultrasound alone can screen for and confirm TP fractures [44]. For cortex‑dependent lesions, ultrasound may even yield more decision-direct information than CT in selected clinical scenarios [45]. Recently, an ultrasound‑only case series successfully detected isolated lumbar TP fractures using direct cortical discontinuity and SDP reproducibility as key diagnostic criteria [46].

The proposed pathophysiology of this patient's condition is a noteworthy aspect of this case and offers a valuable clinical lesson in kinetic chain evaluation. We hypothesize a biomechanical cascade: the initial isolated right sixth rib fracture altered the mechanics of the thoracic cage and the synchronous movement of the lumbar-pelvic rhythm [47,48]. This created an aberrant, repetitive stress load on the lumbar spine, particularly during trunk rotation, with forces likely concentrated at the L2 level. The L2 transverse process, serving as a critical lever arm and attachment point for the psoas major and quadratus lumborum, succumbed to this stress, resulting in a fatigue fracture [11,12,14]. This hypothesis underscores the paramount importance of a holistic evaluation. Clinicians must not view subsequent complaints in isolation but rather consider the entire kinetic chain, especially when initial treatments for an obvious injury fail, and new, persistent pain emerges in a different location [48-50]. This mechanism is substantiated by other cases, such as a report of a lumbar transverse process stress fracture in an elite rower, illustrating how repetitive trunk rotation and high‑load activities can concentrate abnormal stress on the TP [51].

A key technical aspect of our approach worth highlighting was the strategic use of patient positioning. During the diagnostic phase, the patient was placed in a prone position. This provided stability for a bilateral comparative examination and allowed for effective SDP to precisely localize the painful fracture site. For the therapeutic hydrodissection procedure, however, we repositioned the patient into a lateral decubitus position. This was not merely for comfort but a critical maneuver to optimize the acoustic window and, most importantly, to align the needle trajectory parallel to the target fascial planes of the intertransversarii muscles. This tailored positioning enhanced the safety profile of the procedure by avoiding vulnerable neurovascular structures and ensured the injectate could be accurately deposited to achieve effective hydrodissection. This deliberate change in positioning underscores the importance of adapting ultrasound-guided techniques to the specific demands of both diagnosis and intervention.

This report introduces a combined 'image-and-intervene' paradigm as a novel management strategy for chronic, neglected TP fractures. The diagnostic phase leveraged musculoskeletal ultrasound not only for static anatomical identification but, crucially, for dynamic functional assessment via Sonoguided Digital Palpation (SDP). This allowed precise localization of the pain generator and established its clinical relevance. Critically, the dynamic assessment confirmed the absence of gross instability, ruling out the need for surgical stabilization and instead confirming a primary soft tissue etiology for persistent pain. The immediate transition to a therapeutic intervention was therefore justified by the sono-clinical diagnosis of soft tissue entrapment, with fibrosis being the suspected underlying pathology, surrounding the fracture site, which was the source of SDP over the left L2 TP and the associated right lumbar and groin pain. Ultrasound-guided hydrodissection was performed to address the underlying pathophysiology, specifically, the suspected fibrosis and neurovascular entrapment surrounding the left L2 TP, by mechanically dissecting the adherent tissue planes. The use of D5W as the injectate may have provided benefits beyond simple hydrodissection, potentially inducing a regenerative response through neuromodulation and pro-healing stimulation [38,39]. This seamless diagnostic-therapeutic approach resulted not only in rapid pain relief and functional restoration but also appeared to facilitate an environment conducive to bony healing.

A critical element of this paradigm is the anatomically grounded rationale for hydrodissection. The intertransversarii muscles intimately surround the TP and contain mixed peripheral nerves and vessels [52,53]. A chronic fracture can lead to local inflammation, fibrosis, and scar tissue formation, potentially entrapping these neurovascular structures and creating a persistent source of nociception and referred pain [9,10,54]. While chronic fibrotic tissue may not always have a distinct sonographic signature, its functional consequence, restricted gliding between tissue planes, can be inferred during dynamic assessment. Hydrodissection utilizes fluid pressure to mechanically separate these adhered tissues, release entrapment, improve local perfusion, and disrupt pain-generating cycles [55,56]. The injectate, in this case D5W, is also proposed to provide a neuromodulatory effect, potentially decreasing neurogenic inflammation and hyperexcitability of nociceptors. The immediate and profound (80%) pain relief following the procedure strongly supports the notion that the pain was maintained not by the stable fracture itself, but by the surrounding soft tissue and neural entrapment. This explains the complete failure of previous treatments like intercostal nerve blocks and TPVB, which targeted different neural structures, and medications that did not address the mechanical entrapment. This pathophysiology-focused rationale provides a clear basis for targeting the intertransversarii fascial plane [33]. In our protocol, ultrasound‑guided hydrodissection specifically addressed these suspected fibrotic adhesions along the intertransversarii fascial plane, while low‑concentration dextrose exerted perineural neuromodulatory effects, together reducing mechanical entrapment and nociceptive sensitization [57].

This case has several limitations that must be acknowledged. First, the definitive diagnosis was based on radiographs and ultrasound without CT confirmation, which remains the gold standard. However, the strong clinical correlation with examination and the classic imaging findings on two independent modalities (X-ray and US) make the diagnosis highly probable. Second, this is a single case report. While the results are exceptionally promising, larger case series or controlled studies are necessary to validate the efficacy, safety, and reproducibility of this technique across a broader patient population. Finally, the injectate used was D5W, which is believed to have both mechanical and pharmacologic effects. Therefore, the outstanding long-term outcomes observed cannot be attributed solely to the mechanical effect of hydrodissection; the neuromodulatory effect of dextrose likely played a significant role, and the individual contribution of each mechanism remains unclear [36,37].

Despite these limitations, this case highlights an exceptionally valuable and innovative clinical approach. It demonstrates that diagnostic musculoskeletal ultrasound is a powerful, accessible, and dynamic tool for evaluating chronic musculoskeletal pain of bony origin that evades conventional diagnosis. Moreover, it proposes ultrasound-guided hydrodissection as a potent, minimally invasive, and targeted treatment for chronic pain secondary to neglected TP fractures by directly addressing the surrounding soft tissue and neural pathology that perpetuates the pain state.

## Conclusions

In conclusion, this report illustrates that neglected lumbar transverse process fractures can be a hidden and debilitating source of chronic low back and referred pain. A high index of clinical suspicion, particularly when pain persists or presents in a complex referred pattern after trauma, is crucial. Diagnostic musculoskeletal ultrasound offers a reliable, dynamic, and accessible method for identifying these fractures when they are missed by conventional radiographic workup, with Sonoguided Digital Palpation (SDP) providing critical functional correlation. Furthermore, ultrasound-guided hydrodissection of the intertransversarii muscles presents a novel and highly effective treatment strategy. This technique targets the associated soft tissue fibrosis and neural entrapment that often maintain pain long after the initial fracture has stabilized. The presented "image-and-intervene" paradigm achieved significant and sustained pain relief and functional recovery, advocating for its consideration in the management algorithm for similar challenging chronic pain cases refractory to standard treatments. Future controlled studies are warranted to further validate this approach.
